# Spontaneous Rupture of the Patellar Tendon and the Contralateral Quadriceps Tendon, Associated with Lupus Erythematosus: Analysis of the Literature

**DOI:** 10.1155/2011/569363

**Published:** 2012-01-11

**Authors:** Efthimios J. Karadimas, Dimitrios Kotzamitelos, Despoina D. Kakagia, Athanasios Hatziyiannakis

**Affiliations:** ^1^Department of Orthopaedic Surgery, Alexandroupolis University Hospital, 68100 Alexandroupolis, Greece; ^2^Department of Plastic Surgery, Democritus University of Thrace, Dragana, 68100 Alexandroupolis, Greece

## Abstract

Bilateral rupture of the patellar tendon is a rare injury. A case of a 67-year-old man with systemic lupus erythematosus under corticosteroid treatment for the last 10 years, who sustained spontaneous rupture of the patellar tendon and the contralateral quadriceps tendon, is herein presented. The patient was operated bilaterally, had an optimal outcome considering his age and the comorbidities, and was followed up for 24 months.

## 1. Introduction

Isolated quadriceps tendon ruptures tend to occur in patients over 40 years of age, whereas isolated patellar tendon ruptures occur at younger ages. Simultaneous ruptures of the patellar or quadriceps tendons are uncommon. Only few cases have been reported in the literature [1, 2], and are usually secondary to chronic systemic disease such as gout, rheumatoid arthritis, lupus erythematosus, renal disease, hyperparathyroidism, or prolonged corticosteroid treatment [2–8].

 The aim of our study is to present a case of simultaneous rupture of the patellar tendon and the contralateral quadriceps tendon associated with systemic lupus erythematosus.

## 2. Case Report

**Figure 1:**
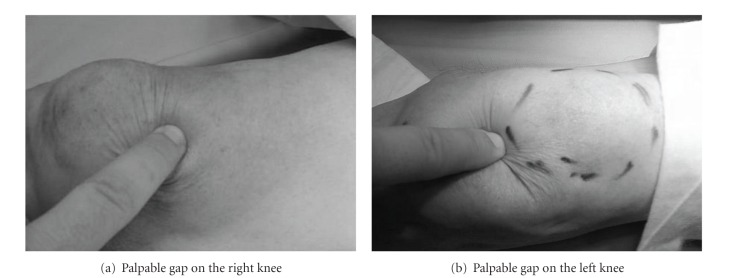


A 67-year-old male was referred to the emergency department due to inability of walking. No accident, fall, or direct trauma was reported. The clinical examination revealed an extended hematoma over the left knee and a less notable hematoma on the right knee. Complete incapability of the extensor mechanism on the left knee and luck of full extension on the other side were also evident. There was palpable discontinuation of tendon, on both sides, but mainly on the right (Figures 1(a) and 1(b)). As there was not any history of trauma, possible bilateral ligament rupture due to an underlying systemic disease was suspected. Patient's history confirmed systemic lupus erythematosus under corticosteroid therapy for the last 10 years.

### 2.1. Preoperative

The patient was admitted, and a supportive brace was applied bilaterally. A specialist rheumatologist examined the patient and adjusted the doses of intravenously administered steroids preoperatively.

**Figure 2:**
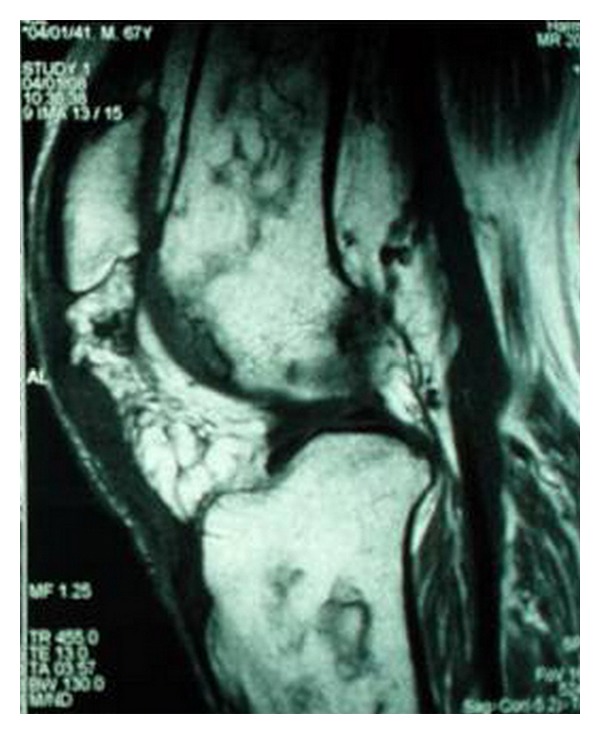
MRI of the left knee reveals complete rupture of the patellar tendon and patella alta.

 The following radiographic evaluation revealed patella alta, which was in agreement with the complete patellar tendon rupture on the left knee, but not on the right side, in which partial quadriceps tendon rupture was suspected. A magnetic resonance imaging (MRI) was performed and confirmed the initial speculations (Figure 2). The report excluded any other injury, as meniscus and cruciate ligaments were found intact.

### 2.2. Treatment

After admission and having signed an informed consent form, the patient underwent reconstruction of the extensor mechanisms of both knees.

**Figure 3:**
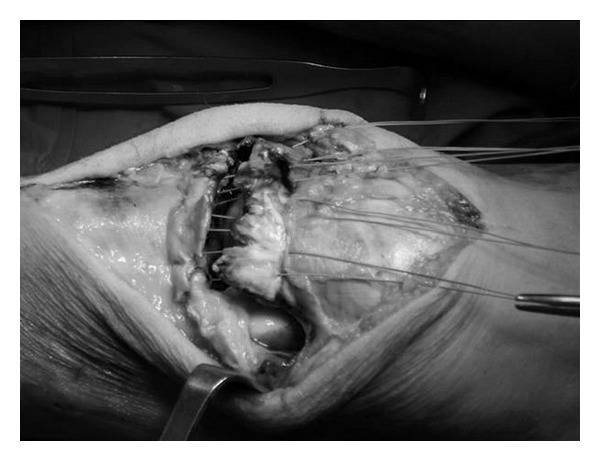
Nonabsorbable sutures through the ends of the ruptured patellar tendon in the left knee.

**Figure 4:**
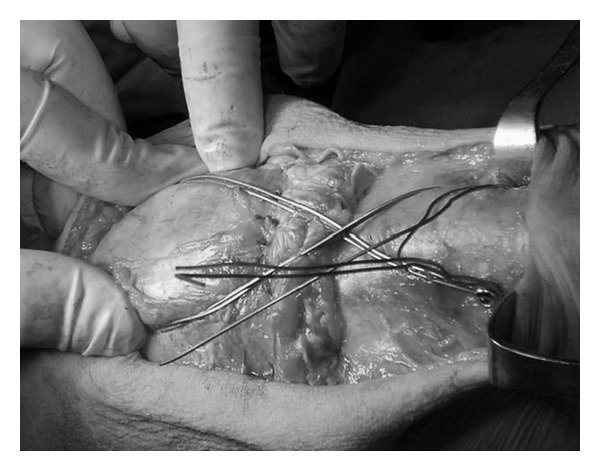
Tension band wire through tibial tuberosity for reinforcing the sutures.

 A tourniquet was used during surgery. On the left side it was inflated at 250 mm Hg for 60 min. Through median longitudinal incision, the two parts of the ruptured tendon were identified; thus, complete rupture of the patellar tendon was confirmed and was reconstructed with the use of non absorbable sutures (Ethibond no. 2) (Figure 3). A 4.5 mm screw was inserted in the tibial tuberosity, and the sutures were reinforced by a metallic wire tension band (Figure 4). The tourniquet was deflated, and meticulous heamostasis was applied. Interrupted sutures were used, and a drain was applied.

**Figure 5:**
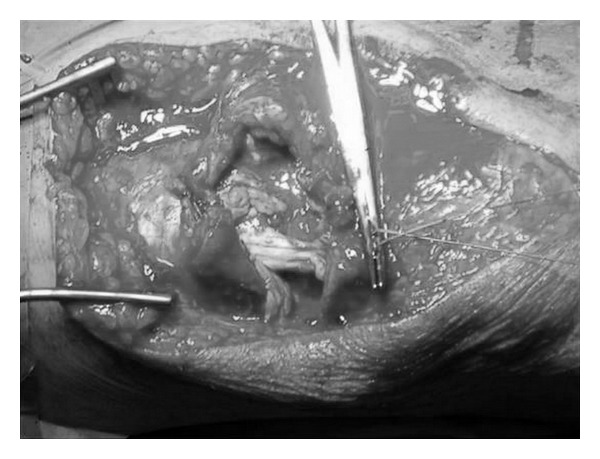
Reconstruction of the partial rupture of the quadriceps tendon in the right knee with nonabsorbable sutures.

 The tourniquet was then inflated above the right knee at 250 mm Hg for 30 min. Using a similar approach centered to the suprapatellar area, a partial quadriceps tendon tear was identified involving almost 75% of the tendon. The rupture was similarly repaired with nonabsorbable sutures (Figure 5). The tourniquet was deflated and meticulous haemostasis was applied. Interrupted sutures were used for skin reapproximation without insertion of a drain.

### 2.3. Postoperatively

Postoperatively, back slab cast was applied to facilitate wound inspection and prohibit knee flexion. The sutures were removed after two weeks and the skin incision healed nicely. At this time, a full cast was also applied with both knees in extension for 8 weeks.

The patient was encouraged to start isometric quadriceps and straight leg rising exercises during the first postoperative week. Partial weight bearing was allowed at four weeks postoperatively with the use of zimmer frame. The patient was reviewed at eight weeks and was referred to the physiotherapy department after removal of plasters. The rehabilitation program included quadriceps strengthening and range of motion exercises. Full weight bearing walking was allowed at that time, according to the patient's comfort.

The patient was followed up regularly due to the rarity of the injury. Although he attended the 6-month appointment using a crouch “for safety,” he was able to walk without any assistance, having nearly full range of motion on both knees. A small degree of atrophy was observed on the left quadriceps compared to the right side, and the patient was advised to have another session of physiotherapy.

After 9 months, the patient was walking unassisted and had nearly full range of motion on both sides. He regained strength on his left quadriceps. Some stiffness during bedding on the left side was considered to be associated with the arthritis previously diagnosed. The patient was satisfied and returned to his daily activities without any restriction.

**Figure 6:**
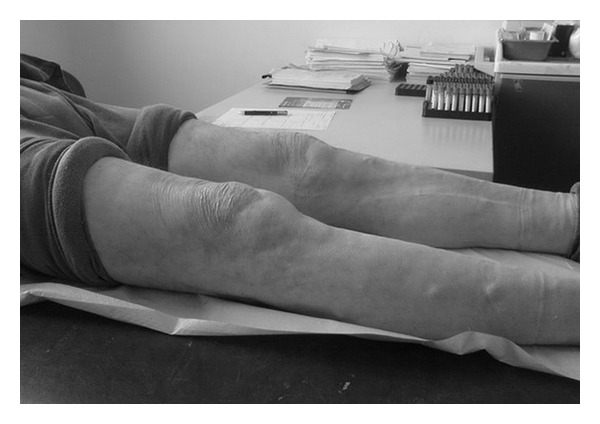
Full extension of both knees in 2-year followup.

**Figure 7:**
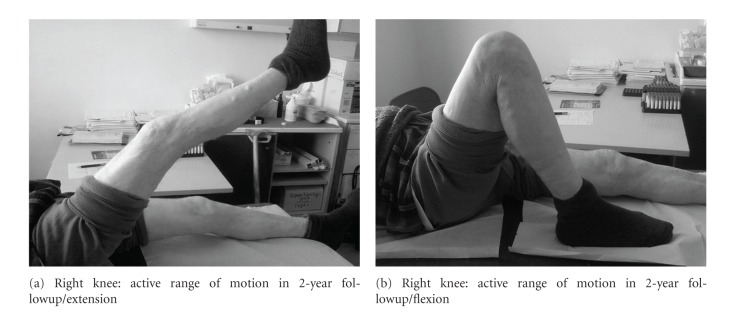


**Figure 8:**
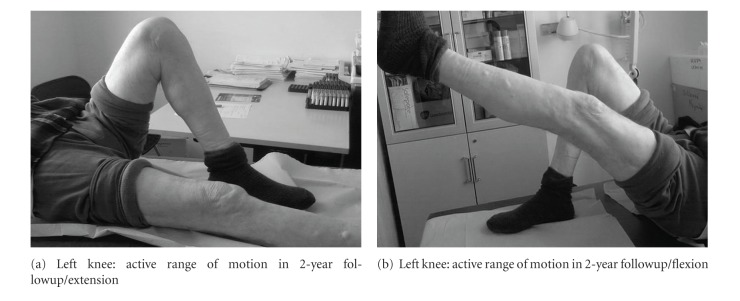


 Two years postoperatively the patient was still walking unassisted and presented a nearly full range of motion in both knees (full flexion bilaterally and 100° on the right and 110° on the left) (Figures 6, 7(a) and 7(b), 8(a) and 8(b)). The Oxford Knee Score was 15 for both knees, an excellent score for the patient's age.

## 3. Discussion

Patella is considered to be the weakest part of the extensor mechanism. People younger than 60 years are by 50–60% more likely to sustain a fracture than a tendon rupture [9].

In younger people, rupture of the patellar ligament at the bone-ligament junction is more frequent than at the tibial tuberosity [10]. The most common mechanism for this type of injury is usually forceful contraction of the quadriceps with the knee partially flexed and against firm resistance [10].

Isolated ruptures usually occur after trauma between the 6th and 7th decades of life [11]. According to Rasul and Fischer, ruptures are classified according to site as musculotendinous, midtendinous, or at the tendon-bone junction [11].

The pathogenesis of major tendon ruptures is not clear. In cases of bilateral ruptures, apart from mechanical factors, investigation for existing comorbidities, such as gout, rheumatoid arthritis, lupus erythematosus, renal disease, hyperparathyroidism, or prolonged corticosteroid treatment is essential [2–8].

In the literature only few cases of bilateral rupture of the quadriceps tendon and the contralateral patellar ligament have been reported [3–5, 12, 13]. Loehr and Welsh [3] analyzed 3 cases of spontaneous tendon ruptures around the knee in patients with chronic renal failure and underlined the importance of the systemic disease contribution in relation to the severity of the trauma. One of those patients was treated for spontaneous patella tendon and contralateral quadriceps ligament rupture, with an end-to-end suture repair of the tendons. A cylinder cast was applied for 6 weeks postoperatively. Six months later the patient had regained full motion and had returned to work.

Other authors [4] reported a case of simultaneous quadriceps and contralateral patellar tendon rupture in a former power lifter who had been treated with several steroid injections in his shoulders in the past but had never been on systemic steroids nor has he had any knee injections. He underwent primary reconstruction with absorbable sutures. Postoperatively cylinder plaster was applied for 48 h at the knee with the quadriceps tendon rupture and for two weeks at the knee with the patellar tendon rupture. A continuous passive motion machine was used to mobilize each knee, once plasters were removed. The patient regained full range of motion bilaterally five and a half months postoperatively.

In 2003, Rogers et al. [5] presented a case of quadriceps tendon rupture with contralateral patellar tendon and emphasized the importance of limb positioning and the degree of knee flexion at the time of injury.

Papanikolaou et al. [12] reported a case of bilateral patellar tendon rupture in a 27-year-old woman as a result of a simple fall. The patient had been diagnosed to have systemic lupus erythematosus (SLE) since the age of 10 and had been on systemic corticosteroid therapy. Apart from osteoporosis treatment, she was not receiving any other medication. Absorbable sutures and reinforcing cerclage wire attached to a screw through the tibial tuberosity were used to reconstruct the ruptures. Functional knee braces locked in extension were applied postoperatively. Physical therapy began immediately with gentle passive motion and isometric exercises out of the brace and no weight bearing. At six months of followup, the patient had achieved full range of motion of the right knee and full extension and 110° flexion of the left knee and was able to walk unassisted.

Other authors [13] analyzed a case of a patient who sustained spontaneous and simultaneous quadriceps, and contralateral patellar tendon rupture. The patient was suffering from chronic renal failure on hemodialysis and from tertiary hyperparathyroidism. The patient had not sustained any direct nor indirect trauma. In both knees the authors performed a reconstruction with absorbable sutures through patellae drill holes according to Krakow's suture technique. Cylinder casts were applied for 5 weeks, followed by a brace for 60 days. At twelve months of followup, the patient reported no pain. The active full extension was possible on both knees, and the bilateral active flexion was of 110°.

Reviewing the published data, it is evident that in our case the patient reached the expected range of motion. Some stiffness on the right knee is considered to be related to the already existing osteoarthritis. Regarding postoperative care, all but one author preferred to protect their reconstruction by using casts and immobilization was followed by physiotherapy.

## 4. Conclusion

Spontaneous rupture of the patellar or quadriceps tendon around the knee is rare, and bilateral ruptures are even more infrequent. In patients with systematic diseases or prolonged corticosteroid treatment, who report incidental knee pain or after minor trauma, possible tendon rupture should be suspected. Early diagnosis and treatment are associated with optimal functional outcome. Surgical management followed by immobilization is the only treatment option and allows for early return of patients to their normal daily activities.

## References

[1] Murphy KJ, McPhee I (1965). Tears of major tendons in chronic acidosis with elastosis. *Journal of Bone and Joint Surgery*.

[2] Wener JA, Schein AJ (1974). Simultaneous bilateral rupture of the patellar tendon and quadriceps expansions in systemic lupus erythematosus. A case report. *Journal of Bone and Joint Surgery A*.

[3] Loehr J, Welsh RP (1983). Spontaneous rupture of the quadriceps tendon and patellar ligament during treatment for chronic renal failure. *Canadian Medical Association Journal*.

[4] Munshi NI (1996). Simultaneous rupture of the quadriceps tendon with contralateral rupture of the patellar tendon in an otherwise healthy athlete. *British Journal of Sports Medicine*.

[5] Rogers A, Rix S, Kulkarni R (2003). Simultaneous rupture of a patellar tendon and contralateral quadriceps tendon in a healthy individual. *Orthopedics*.

[6] Morgan J, McCarty DJ (1974). Tendon ruptures in patients wit systemic lupus erythematosus treated with corticosteroids. *Arthritis and Rheumatism*.

[7] Chen CH, Niu CC, Yang WE, Chen WJ, Shih CH (1999). Spontaneous bilateral patellar tendon rupture in primary hyperparathyroidism. *Orthopedics*.

[8] Clark SC, Jones MW, Choudhury RR, Smith E (1995). Bilateral patellar tendon rupture secondary to repeated local steroid injections. *Journal of Accident and Emergency Medicine*.

[9] Scuderi C (1958). Ruptures of the quadriceps tendon. Study of twenty tendon ruptures. *The American Journal of Surgery*.

[10] Nanninga AJ, Josaputra HA (1987). Tibial tuberosity fracture in adolescents. Report of a case and review of the literature. *Netherlands Journal of Surgery*.

[11] Rasul AT, Fischer DA (1993). Primary repair of quadriceps tendon ruptures: results of treatment. *Clinical Orthopaedics and Related Research*.

[12] Papanikolaou A, Charalambides C, Thanassas C (2007). Spontaneous simultaneous bilateral patellar tendon rupture in a systemic lupus erythematosus patient. *Lupus*.

[13] Grecomoro G, Camarda L, Martorana U (2008). Simultaneous chronic rupture of quadriceps tendon and contra-lateral patellar tendon in a patient affected by tertiary hyperparatiroidism. *Journal of Orthopaedics and Traumatology*.

